# The Large ARtery Intracranial Occlusion Stroke Scale: A New Tool With High Accuracy in Predicting Large Vessel Occlusion

**DOI:** 10.3389/fneur.2019.00130

**Published:** 2019-02-19

**Authors:** Simone Vidale, Marco Arnaboldi, Lara Frangi, Marco Longoni, Gianmario Monza, Elio Agostoni

**Affiliations:** ^1^Department of Neurology and Stroke Unit, Sant'Anna Hospital, Como, Italy; ^2^Department of Neurology and Stroke Unit, Niguarda Ca' Granda Hospital, Milan, Italy; ^3^Department of Intensive Care Unit and Emergency Medical Service, Sant'Anna Hospital, Como, Italy

**Keywords:** stroke, pre-hospital scale, large vessel occlusion, thrombolysis, mechanical thrombectomy

## Abstract

**Objectives:** The combination of systemic thrombolysis and mechanical thrombectomy is indicated in patients with ischemic stroke due to a large vessel occlusion (LVO) and these treatments are time-dependent. Rapid identification of patients with suspected LVO also in a prehospital setting could influence the choice of the destination hospital. Aim of this pilot study was to evaluate the predictive role of a new stroke scale for LVO, comparing it to other scores.

**Patients and Methods:** All consecutive patients admitted to our comprehensive stroke center with suspected ischemic stroke were studied with a CT angiography and 5 different stroke scales were applied. The Large ARtery Occlusion (LARIO) stroke scale consists of 5 items including the assessment of facial palsy, language alteration, grip and arm weakness, and the presence of neglect. A Receiving Operating Characteristic curve was evaluated for each stroke scale to explore the level of accuracy in LVO prediction.

**Results:** A total of 145 patients were included in the analysis. LVO was detected in 37.2% of patients. The Area Under Curve of the LARIO score was 0.951 (95%CI: 0.902–0.980), similar to NIHSS and higher than other scales. The cut-off score for best performance of the LARIO stroke scale was higher than 3 (positive predictive value: 77% and negative predictive value: 100%).

**Conclusion:** The LARIO stroke scale is a simple tool, showing high accuracy in detecting LVO, even if with some limitations due to some false positive cases. Its efficacy has to be confirmed in a pre-hospital setting and other centers.

## Introduction

The combination of systemic thrombolysis and mechanical thrombectomy is indicated in patients with ischemic stroke due to a large vessel occlusion (LVO), and this recommendation has been validated by previous randomized controlled trials (1–4). However, because stroke is a time-dependent disease, the benefit of these treatments is highly influenced by rapid identification of symptoms in the pre-hospital setting and concomitant fast transportation to comprehensive centers, which have limited beds and resources. For this reason, the concomitant time sensitivity for both treatments could influence the choice of the destination hospital. In recent years, several scales have also been applied to select patients with suspected stroke due to a large vessel occlusion in a pre-hospital setting. A recent meta-analysis of these studies showed a great heterogeneity of results with the best prediction observed for some scales (5), even if all presented a lower accuracy than the National Institute of Health Stroke scale (NIHSS) (6), and for this reason they presented several limitations in their application in a real-world setting. According to these evidences, the vessel images are mandatory to select patients for mechanical thrombectomy as they detect LVO with higher accuracy.

Aim of this pilot study was to improve the prediction of large vessel occlusion, assessing a new tool called Large ARterial Intracranial Occlusion (LARIO) Stroke Scale.

## Materials and Methods

### Subjects

All patients admitted consecutively to our hospital between April and October 2017 with suspected ischemic stroke and with a brain CT scan excluding hemorrhage were also studied with a CT angiography of extra- intracranial arteries after a given written informed consent. At hospital arrival, different stroke scales have been administered to all patients by a single neurologist with a separate evaluation of a trained nurse using only the new tool. We excluded from this analysis patients with symptoms over 24 h or if the iodinated contrast agent administration was contraindicated (see flow-chart in Supplemental Material). The assessment order of stroke scales was: LARIO, Cincinnati Prehospital Stroke Severity Scale (CPSSS) (7), Los Angeles Motor Scale (LAMS) (8), Vision, Aphasia, Neglect assessment (VAN) scale (9), and NIHSS. For each patient, we registered also gender, age, stroke etiology, and the clinical syndrome, using TOAST and OCSP criteria.

### Tool Assessment

The LARIO stroke scale was designed on the basis of the LAMS, adding “Neglect” as new item to this scale. LAMS was chosen because this tools showed high accuracy to detect LVO in previous studies and a concomitant simple assessment in a pre-hospital setting (5, 8). We added the evaluation of spatial cognition on the basis of our previous findings that showed a higher prediction of LVO using this variable than other items (5). In our scale we evaluated facial palsy, arm weakness, grip weakness, language alteration (aphasia or dysarthria), and finally presence of neglect (reported as an extinction to bilateral simultaneous stimulation in one or more sensory modality or an unrecognized own hand or orientation only to one side of the body). For each item we gave a score of 0 if absent and 1 if present (Table 1). We calculated the score with a possible range between 0 (normal) and 5 (maximum). The training for the nurse was planned using the online software for NIHSS certification with a particular attention to the evaluation of the neglect (www.nihss-neurosapienza.trainingcampus.net/) and it took about 1 week.

**Table 1:** The LARIO Stroke Scale.

**Item**	**Score**
**FACIAL PALSY**	
Normal	0
Present	1
**ARM WEAKNESS**	
No drift	0
Drift or no effort against gravity or no movement	1
**GRIP STRENGTH**	
Normal	0
Reduced or absent	1
**LANGUAGE**	
Normal	0
Changes or global aphasia, or mute	1
**NEGLECT**	
Absent	0
Extinction to bilateral simultaneous stimulation in one or more sensory	1
Modality or an unrecognized own hand or orientation only to one side of the body	

*LARIO, Large ARtery Intracranial Occlusion*.

### Image Protocol and Review

Two expert neurologists with certified vascular and radiological experience reviewed all images of brain CT and CT Angiography. They were blinded to patients' demographic and clinical data and they had not participated to the assessment of the scales. For each image, vessels were evaluated and graded for the presence or absence of a total occlusion and its site (intracranial internal carotid artery, the M1 and M2 tracts of the middle cerebral artery (MCA) and basilar artery).

### Aim of the Study

The principal aim of this pilot study was the validation of a new screening tool detecting LVO in ischemic stroke patients, exploring also its level of accuracy compared with other scales. The secondary aim was to investigate the agreement level of the LARIO scores between the neurologist's and nurse's evaluations.

### Sample Size Calculation

We estimated a sample size for adequate sensitivity and specificity, using the formula:

(1) $$\begin{array}{l} n=\frac{Z_{\frac{\alpha }{2}}^{2}\hat{P}\left(1-\hat{P}\right)}{d^{2}} \end{array}$$

We postulated a sensitivity of about 0.80 and a specificity of 0.90 from previous study concerning the accuracy of LAMS score (5). At the same time, we assumed a marginal error of 0.10 and a prevalence of LVO in ischemic stroke patients of about 0.35–0.40. By these parameters, we obtained the number of 141 as sample size calculation.

### Statistical Analysis

Categorical variables were reported as proportions, while continuous variables were presented as median and interquartile ranges (IQR). We applied the chi-squared and *t*-test to compare categorical and continuous variables between groups. Receiver operating characteristic (ROC) curve analysis was applied to test the discrimination ability and the grade of accuracy of our scale applied by neurologist to predict a LVO, compared to the other tools. Sensitivity, specificity, positive, and negative predictive values were calculated for the new scale at different threshold. The Youden Index was used to calculate the optimal threshold of the LARIO stroke scale. Finally, we compared the sensitivity, specificity, and accuracy of the new scale at predefined score with other scales at prespecified published thresholds. The Cohen's k test was used to verify the degree of agreement between the scores of the LARIO stroke scale performed by the neurologist and nurse. We considered significant a *p*-value < 0.05. All statistical analysis were performed using SPSS software.

## Results

One hundred forty-five qualifying patients with ischemic stroke were selected. The median age was 77 years (IQR: 68–83) with prevalence of males equal to 60.7%. A LVO was detected in 54 patients (37.2%) with prevalence for the MCA of 43% (Table S1). The median score for the NIHSS was 7 (IQR: 4–17), while the LARIO stroke scale had a median score of 3 (1–4). The scores of the other applied scales are presented in Supplementary Material. While in the absence of LVO we observed low median scores of the different scales (from 0 of LAMS to 5 of NIHSS), in patients with LVO, the NIHSS, the LARIO stroke scale, the CPSSS, and the LAMS had, respectively, scores of 18, 4, 3, and 4. Considering the presence of LVO, we observed a significant difference in age (79 vs. 73; *p* < 0.05), but not for gender. Median onset-to-door time was 84 min (58–121) and time between assessment and CTA was 5 min (2–8).

**Figure 1:**
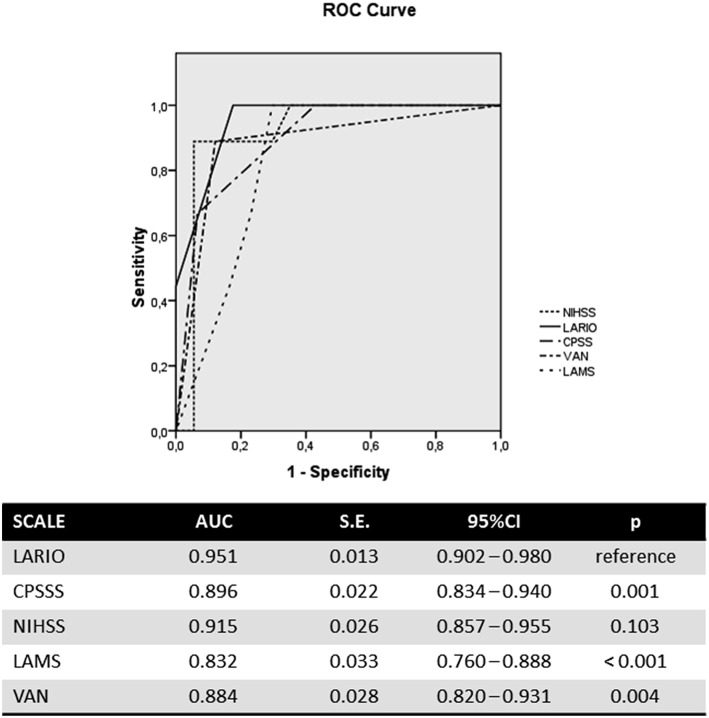
ROC curves comparing different stroke scales.

The LARIO stroke scale showed a similar accuracy to the NIHSS in predicting LVO and a higher accuracy than the other scales. In particular, the area under curve (AUC) for our scale was 0.951 (95%CI: 0.902–0.980), while NIHSS showed an AUC of 0.915 (0.587–0.955). The ROC curve is represented in the Figure 1. The AUC of LARIO stroke scale was higher in the left hemispheric stroke than right cerebral ischemia (0.966 vs. 0.932), counting 36 patients with left stroke due to LVO (54 subjects had not a LVO) and 19 patients with right stroke and LVO (36 subjects had no LVO). However, the findings were significant in both sides. Pairwise comparisons of ROC curves confirmed the superiority of accuracy of NIHSS and LARIO on the other scales and a no-significant difference between the two tools (Table S3). According to the Youden Index, the best performance of the LARIO stroke scale was observed at the score superior to 3. At this criterion we calculated a sensitivity of 1 and a specificity of 0.82. The comparison of accuracy at this level of the LARIO stroke scale with other tools is showed in Table S6. While NIHSS administration was time consuming (mean time: 3.4 min; S.D.: 4.3 min), the administration of the LARIO stroke scale was faster (mean time: 1.2; S.D.: 3.2).

Finally, we observed a high agreement between the neurologist's and nurse's scores of the LARIO stroke scale (Cohen's k: 0.963; S.E.: 0.012; 95%CI: 0.940–0.986; *p* < 0.001).

## Discussion

In this pilot study, we observed that the LARIO Stroke scale at the best-calculated cut-off score has a high sensitivity and high specificity for the detection of LVO. In our trial, the rate of this condition was higher than in other studies, but it is in line with the prevalence of a very recent systematic review (10). However, our observation could also be due to a referral bias into the study. The Youden index showed the best prediction of LVO for a score of more than 3. At this cut-off score, the LARIO Stroke scale showed a significantly higher discrimination of patients with LVO when compared with the other scales, NIHSS excluded. A recent study showed a good identification of LVO patients using the CPSSS, with a ROC of 0.64. In the present study, the CPSSS had a higher value of ROC than the previous one, but lower than the new scale (11). In a previous review, we detected the best accuracy in detecting LVO for VAN, LAMS and NIHSS (5). However, in our sample we observed lower values for sensitivity and specificity for the VAN scale and a significant difference in the comparison of ROC curves, as well as for the LAMS scale. The remaining scale represented by the NIHSS showed similar results to the LARIO Stroke scale in detecting LVO. However, the NIHSS could be too complex for a pre-hospital setting in terms of time consumption and expertise of users. In light of these observations, our new scale appears simple to apply also for non-specialist professionals and with a limited time of application. Even though neglect is probably one of the most complex issues to assess, and one which carries a high chance of wrong interpretation by a non-neurologist, in our study we observed a significant agreement between neurologists' and nurses' evaluations of this condition. According to this observation, specific and effective training concerning the detection of this item could represent the crucial point for a correct assessment of the neglect.

Considering the different territories of LVO, in our pilot study the new scale also showed possible prediction for basilar occlusion because all six patients with this condition had a positive score on the LARIO Stroke scale. However, this observation needs to be confirmed with a greater sample. A limitation of the new tool could be an isolated or combined visual or cerebellar deficit, because these are not included in the assessment of the LARIO Stroke scale. However, basilar occlusion is unlike a condition of an isolated symptom; rather, several signs, and deficits are often reported (12, 13). At the same time, the presence of language and neglect assessments in the LARIO Stroke scale contributed to avoiding a failed detection of an MCA occlusion.

Recent European and American recommendations indicate that clinical screening tools may be introduced to facilitate the selection and triage of patients with suspected LVO who could be directly transferred to a comprehensive stroke center (14, 15). However, as vessel images detect LVO, they cannot be replaced by stroke scales in selecting patients for mechanical thrombectomy. Even if stroke scales showed high accuracy, they would have a significant false positive rate, as shown in the present pilot study (16, 17).

This could be one of the study limitations, but, even though we observed a false positive rate of 18%, no false negative cases were observed. A second limitation could be the setting in which it was applied (in-hospital and not extra-hospital) and the raters that applied the new scale. However, this is a pilot study and we observed a high accordance between scores obtained from neurologists' and nurses' evaluations after a brief training session that could be applied also for volunteers of the emergency medical services. Third, this scale is not able to differentiate ischemic from haemorrhagic strokes, but the direct transfer of severe patients with brain hemorrhage could benefit from a comprehensive stroke center with more facilities than a primary stroke center. Finally, we observed a 37.2% of LVO that indicates a prevalence rate higher than in population-based settings, and this result could be influenced by a referral bias, as previously mentioned.

There are some strengths for this new tool. First, it is quite simple and user-friendly after brief and effective training. Second, our pilot study to validate this scale was prospective with consecutively enrolled ischemic stroke patients. Third, all patients were studied with CT angiography with external validation of imaging.

## Conclusion

Effective pre-hospital triage and organization for severe strokes have to reduce both the symptom onset-to-groin puncture time and hospital-to-hospital transfers. According to these aims, our scale could provide a significant response to the need for the use of an effective tool to optimize the triage of stroke patients suspected of LVO. These findings are preliminary to a new study assessing the feasibility of the LARIO Stroke Scale in a pre-hospital setting, including a larger sample of patients and the application by a wide variety of providers, also in other hospital centers.

## Ethics Statement

The study was part of the routine clinical activity, adding the application of the new scale. Each patient signed a written consent for radiological examination and for anonymous general use of data concerning the hospitalization, according with local governmental acts.

## Author Contributions

SV and EA conceived and designed the study. SV and LF obtained the data for the work. ML analyzed the data. SV, ML, and EA interpreted the data for the work. SV drafted the work and ML and EA revised critically the paper for important intellectual content. All authors approved the final version of the paper to be published and agreed the accountability for all aspects of the work in ensuring that questions related to the accuracy or integrity of any part of the work were appropriately investigated and resolved.

### Conflict of Interest Statement

The authors declare that the research was conducted in the absence of any commercial or financial relationships that could be construed as a potential conflict of interest.
