# Supplementary material for: The Large ARtery Intracranial Occlusion Stroke Scale: A New Tool With High Accuracy in Predicting Large Vessel Occlusion
Source: Front Neurol. 2019 Feb 19;10:130. doi: 10.3389/fneur.2019.00130 (PMC6389631; doi:10.3389/fneur.2019.00130)

Table S1: Radiological and baseline data

| Variable | N. |
| --- | --- |
| Age (mean ± SD) | 75 ± 11 |
| Gender (Male) | 88 |
| Clinical syndrome |  |
| LACS | 39 |
| PACS | 11 |
| TACS | 69 |
| POCS | 26 |
| Etiology |  |
| Large Artery Atherosclerosis | 30 |
| Cardioembolism | 38 |
| Lacunar | 60 |
| Unknown | 17 |
| Other | 0 |
| Site of occlusion |  |
| ICA (left) | 18 (12) |
| MCA- M1 (left) | 24 (6) |
| MCA – M2 | 6 |
| BA | 6 |

Table S2: Median scores of scales

| Scale | Median | IQR |
| --- | --- | --- |
| NIHSS | 7 | 4 – 17 |
| LAMS | 3 | 0 – 5 |
| CPSS | 2 | 1 – 3 |
| LARIO | 3 | 1 – 4 |

Table S3: Pairwise comparisons of ROC curves

|  | CPSSS | LAMS | NIHSS | LARIO SS | VAN |
| --- | --- | --- | --- | --- | --- |
| CPSSS |  | 0.035  (-0.005 – 0.134) | 0.029  (-0.039 – 0.078) | 0.017*  (0.022 – 0.089) | 0.023  (- 0.033 – 0.056) |
| LAMS | - |  | 0.028*  (0.028 – 0.139) | 0.028*  (0.065 – 0.174) | 0.037  (- 0.021 – 0.126) |
| NIHSS | - | - |  | 0.022  (- 0.007 – 0.079) | 0.019  (- 0.007 – 0.069) |
| LARIO SS | - | - | - |  | 0.023*  (0.022 – 0.112) |

* p < 0.05; (Standard Error; 95% Confidence Interval)

Table S4: Median scores of the scales for subgroups of patients with or without LVO

|  | LVO | | No LVO | |
| --- | --- | --- | --- | --- |
| Scale | Median | IQR | Median | IQR |
| NIHSS | 18 | 16 –21 | 5 | 4 – 7 |
| LAMS | 4 | 3 – 5 | 0 | 0 – 3 |
| CPSSS | 3 | 2 – 3 | 1 | 1 – 2 |
| LARIO | 4 | 4 –5 | 2 | 1 –34 |

Table S5: Sensitivity, specificity, positive and negative predictive values for LARIO stroke scale at different intervals of score

| Score | Sensitivity | Specificity | PPV | NPV |
| --- | --- | --- | --- | --- |
| > 0 | 1 | 0.17 | 0.41 | 1 |
| > 1 | 1 | 0.45 | 0.52 | 1 |
| > 2 | 1 | 0.63 | 0.62 | 1 |
| > 3 | 1 | 0.82 | 0.77 | 1 |
| > 4 | 1 | 1 | 1 | 1 |

Table S6: Comparison of sensitivity, specificity, positive and negative predictive values according to different thresholds of LARIO stroke scale, NIHSS, CPSSS, LAMS, and VAN scale

| Scale | Sensitivity | Specificity | PPV | NPV |
| --- | --- | --- | --- | --- |
| LARIO > 3 | 1 | 0.82 | 0.77 | 1 |
| CPSSS > 2 | 0.66 | 0.94 | 0.88 | 0.83 |
| NIHSS > 9 | 0.89 | 0.83 | 0.76 | 0.92 |
| LAMS > 3 | 0.34 | 0.56 | 0.32 | 0.57 |
| VAN positive | 0.89 | 0.88 | 0.81 | 0.93 |

Figure S1: Flow-chart for included/excluded patients

**161 ischemic stroke patients**

8 patients with symptoms over 24 hours

4 patients with severe renal failure

2 poliallergic patients

2 patients withdrawn consensus

145 patients

147 patients

Figure S2: Youden Plot for inter-rater variability assessing the LARIO stroke scale


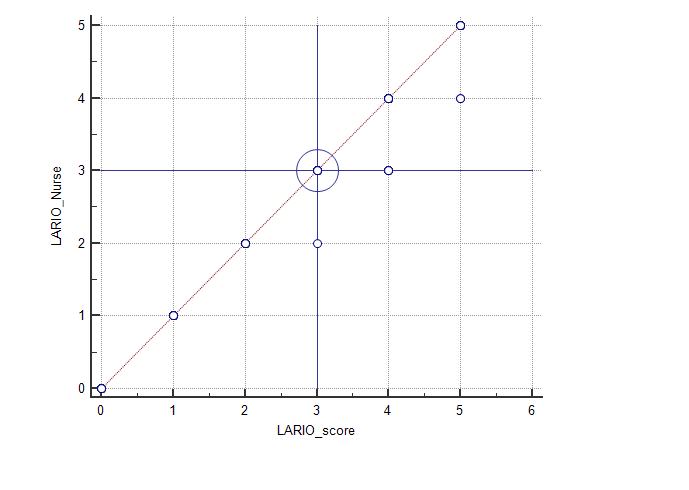

Supplement: Supplementary file 1 [file Data_Sheet_1.docx]
